# Solutions and Deposits of Mineral Springs

**Published:** 1858-01

**Authors:** 


SOLUTIONS AND DEPOSITS OF MINERAL AND THERMAL SPRINGS. — The water of springs, which, in its subterranean course, had abstracted from the soil and rocks a great variety of substances, and had been penetrated with different gases, parts with some of these, often in considerable quantities, after its issue from the earth and exposure to the atmosphere. In this way, compensation is made by deposits on the surface for the materials which had been carried off by solution in the interior; and the water gives evidence above of the company which it keeps under ground.

The solvent power of water on the different earths, as they are combined in rocks, is much greater than is generally imagined; more especially when it is aided by an elevated temperature, and, also, by the addition of carbonic acid. Drs. W. B. and R. B. Rogers, in experiments made for the purpose, found that by prolonged digestion of the pulverized mineral, such as hornblende, epidole, mesotype, etc., in simple or carbonated water, an appreciable quantity of their constituent ingredients—lime, magnesia, oxide of iron, alumina, silica, and alkali—amounting sometimes to one per cent. of the whole mass, was dissolved. The experimenters found, contrary to common belief, that the carbonate of magnesia is much more soluble than carbonate of lime. They point out the readiness with which magnesia and the calcareo-magnesian silicates yield to the decomposing and dissolving action of carbonated and even simple water. M. Damour, in a paper on the *Thermal Springs of Iceland*, attributes the introduction of silex and alkalies into these waters to the decomposing action of pure water, of an elevated temperature, and, under considerable pressure, in trachytic rocks which serve as their recipient. To the same purport, as illustrating solution and deposit by springs, are the experiments of M. H. de Senarmont, on the artificial formation, by the moist method, of some species of minerals, which may be produced in thermal springs under the combined influence of heat and pressure. Carbonate of magnesia may be obtained by a double decomposition, at a temperature of 320° Fahr., of carbonate of soda and sulphate of magnesia. Carbonate of iron may be the product of the action of carbonate of lime on the protochloride of iron, at temperatures of 273° to 356° Fahr., kept up from twelve to eighteen hours; also of the double decomposition, at 302°, of sulphate of iron by the bi-carbonate of soda; and of other double decomposition not necessary to be specified at this time. A great many metallic sulphates are easily prepared, as M. de Senarmont shows, by the moist method in the laboratory. He has procured a bi-sulphuret of iron by double decomposition, at 165° Fahr., of the protochloride of iron and the per-sulphuret of potassium.

There are differences in the readiness of precipitation of sulphurets and carbonates in formations, so that the first are more readily precipitated, and hence form a lower deposit than the carbonates, from waters saturated with the respective gases. As illustrating the changes by double decomposition produced by certain combinations, we might suppose, for example, that waters charged with chlorides of calcium, of magnesium, and iron, should mix, under certain conditions of temperature, and consequently of pressure, with carbonates containing more or less sulphate of soda, and give rise at the same time to deposits of magnesia and lime, and anhydrates of red oxide of iron, and rock-salt. Metalliferous veins are, in the opinion of M. de Senarmont, due to thermal springs. On this point we shall have something to say at another time.

The most frequent and largest deposits are calcareous, which, becoming hard by time and exposure to the air, form extensive quarries, from which the materials for building are procured to any conceivable amount. These may come both from hot and from cold springs, as in the famed rock named *travertin*, by the Italians; out of which the temples, amphitheatres, and triumphal arches of ancient, and the palaces and churches of modern, Rome are built. Of similar material and formation are the deposits which were used in the construction of the temples of Pæstum. Round Guancavelica, in South America, there are many considerable hot springs which yield the calcareous matter that had been held in solution. So strong is the impregnation of the spring-water, that the inhabitants receive it in square boxes or moulds, which are filled in a few days with the matter it deposits, and the blocks thus formed soon become fit material for building, and are used for this purpose.

At the Baths of San Vignone, in Tuscany, at a short distance from Radacofini, and only a few hundred yards on the high road between Sienna and Rome, we see, on a large scale, the petrifying action of carbonated waters, in regions which lie near to the active sources of volcanic disturbance. One of the most striking examples of the rapid precipitation of lime from thermal waters occurs in the hill of San Vignone. The spring issues from near the summit of a rocky hill, about one hundred feet in height. The fundamental rock from which the spring issues is a black slate, with serpentine, belonging to the older Apennine formation.

The water is hot, has a strong taste, and, when not in very small quantity, is of a light green color. So rapid is the deposit near the source, that in the bottom of a conduit-pipe for carrying off the water to the baths, and which is inclined at an angle of 30°, half a foot of solid travertin is formed every year. A more compact mass of rock is produced where the water flows freely; and the precipitation in winter, when there is least evaporation, is said to be more solid, but less in quantity by one-fourth, than in summer. The rock is generally white; some parts of it are compact, and ring to the hammer; others are cellular, and with such cavities as are seen in the caries of bone; they are found in the silicous mill-stone of the French basin. A portion below the village of San Vignone consists of incrustation of long, vegetable tubes, and may be called *tufa.*

A large mass of *travertin* descends the hill from the point where the spring issues, and reaches to the distance of about half a mile east of San Vignone. The beds take the shape of the hill at about an angle of 6°, and the places of stratification are perfectly parallel. One stratum, composed of many layers, is of a compact nature, and fifteen feet thick; it serves as an excellent building-stone. Another branch extends to the west for two hundred and fifty feet in length, and of varying thickness, but sometimes two hundred feet high. It is then cut off by the small river Orcia, which undermines it. The quantity of travertin thus collected is insignificant compared with that which has been carried to the sea since the time when it began to flow.

*The Baths of San Filippo*, as they are called, furnish one of the most noted examples of deposit. Not many miles distant from the hill of San Vignone, on another, the summit of which is about three miles distant, are the celebrated baths of San Filippo. There are three warm springs here, containing carbonate and sulphate of lime, and sulphate of magnesia. The water which supplies the baths falls into a pond, where it has been known to deposit a solid mass *thirty feet thick* in about *twenty years.* A manufactory of medallions, in basso-relievo, is carried on in these baths, by causing a deposit of the water, freed from travertin and crystals of sulphate of lime, in moulds. The result is a beautiful cast of the figures formed on the mould. A hard stratum of stone, about a foot in thickness, is obtained from the waters of San Filippo in four months. The mass of calcareous deposit which descends the hill is a mile in breadth, in some places attaining a thickness of two hundred and fifty feet at least. The whole length it might have reached it is impossible to conjecture, as it is cut off, like the travertin of San Vignone, by a small stream, where it terminates abruptly.

The *spheroidal structure* in the travertin of this region, like that of the cascade of Tivoli, is of peculiar interest to the geologist. The lamination of some of these concentric masses is so minute that sixty may be counted in the thickness of an inch. Sections are, notwithstanding, exhibited of what might be called perfect spheres.

Other examples of calcareous deposit on a large scale and with picturesque effect—forming in one place lofty mounds, and in another a series of terraces—are exhibited at the thermal springs of Hieropolis, and of Singerli in Asia Minor; also in those of Hammam-mes Koutin in Algeria, and also in New Zealand. From the springs near Lake Oroomiah is deposited the translucent Tabreez marble or alabaster. Vegetable and other bodies immersed for a time in the water of springs thus impregnated receive a calcareous coating and become petrified. So complete is the conversion of a plant, for example, into stone, in this process of petrifaction, by which all its tissues are saturated and every interstice filled up, that, but for its figure and the filaments and lines marking its original structure, it would be no longer recognizable as having ever belonged to the vegetable kingdom.

Hieropolis was the most noted of the cities of Asia Minor for the profusion of its thermal waters and the splendor and embellishments of its baths. It was called by distinction the "Bath;" and the city itself, no doubt with something of Oriental hyperbole, was described as "the most splendid." The calcareous deposit from these springs is so great as to have formed the hill on which the remains of the city stand; and their track, which might be called a petrified stream, is marked by similar deposits, extending several hundred feet into the plain beyond. As this flowed down the hill, the waters met with just interruption enough to form, by their deposits, a succession of terraces, suggesting the idea of an amphitheatre. In another direction, where the side of the hill is more precipitous, the eye is arrested by one continuous snow-white street, which bears the appearance of a solid cataract sixty to seventy feet high and a hundred and fifty feet wide. One traveller (Hamilton) tells us that in front of what was once the theatre, at the bottom of a large pool into which the waters of the springs flow, he could distinguish traces of a colonnade of beautifully fluted columns, as perfect as when they were first thrown down. They probably, he adds, once surrounded the sacred fountain, and now look like the ruins of a naiad's palace. The striking appearance presented by these calcareous deposits has caused the Turks to confer on Hieropolis the name of *Pambuk-kelesy*, cotton castle.

The Tabreez marble is deposited from warm carbonated springs on the east side of Lake Oroomiah, which is about forty-five miles distant from Tabreez, the capital of Azerbijan, the northwesternmost province of Persia, and not far from the Caspian Sea. It is the most important commercial city of Persia, and the great mart for European merchandise. The waters of the lake are among the most saline in the world, containing, as they do, substances coming under this head to the amount of 22·5 per cent. The chief calcareous deposits occur within half a mile of the town of Maraha, which is ten miles east of the lake. The Tabreez marble, or alabaster it might be termed, is of a yellowish, running into a light blue color, very compact, and so translucent that it is used, when cut into thin slices, as a substitute for glass in the windows of baths and other edifices. Cold and thermal carbonated springs, containing both lime and soda, abound on both sides of Lake Oroomiah. Sulphur and salt springs are also met with in this region. The Rev. Mr. Perkins passed one great quarry within a few rods of a spring from which vast quantities of this marble have been taken. This quarry is but little above the level of the lake, and is about half a mile distant from its eastern shore.

Siliceous deposits, implying the previous solution of silex, are chiefly seen in volcanic districts, as in Iceland, St. Michael's, one of the Azores, etc. Pure water, at the boiling point, (212° Fahr.) acting on palagonite, the chief constituent of which is silex, dissolves this latter, or rather the silicic acid, as Bunsen ascertained, and also potash and soda. When the water was heated with carbonic acid and allowed to act on pulverized palagonite, all the constituents of the latter, with the exception of alumina and oxide of iron, were dissolved in the form of bi-carbonates. M. Eugene Robert found, that on comparing a specimen of unaltered phonolithe of the mountain of Langarfrall, not far from the great Geyser, with another that had been exposed to degradation and decomposition, the former contained 72·3 per cent. of silex, and the latter only 65·8 per cent. The difference shows the proportion of silex abstracted and dissolved. This last process is accomplished either by the high temperature of the water, which is sometimes as high as 124 C.=255° Fahr., as in the Geysers, or rather, as M. Dumas thinks, the change is produced by the repeated action of the watery vapor, which escapes from the spring against the heated sides of the reservoir. It has been suggested that alumina may be liberated from argillaceous rocks by a similar process. A still larger solution of silex in water is obtained by the intervention of an alkali, commonly soda, more particularly when a high temperature is not necessary. The volcanic rocks near the Geysers are chiefly silicates.

Sometimes the deposit consists of a mixture of a carbonate and earthy silicate, with oxide of iron, as in that from the spring of St. Allyre, at Clerment. It forms a large part of the soil in one quarter of the city, and has furnished the material for a natural bridge over a small river close by. In Iceland and St. Michael's, petrifactions of vegetable products, formed by the incrustations of siliceous matter, are readily seen in all stages, from the soft state to a complete conversion into stone. The siliceous formations by deposits on the surface of the earth are inconsiderable, but it has been conjectured that a much larger quantity is furnished by springs under the ocean, and that this latter is overlaid far and wide by layers of nodules of silex, and thus interstratified with shelly and calcareous deposits which may be forming there, or with matter derived from wasting cliffs or volcanic ejections. The water of the thermal siliceous springs in Iceland, as at the Geysers more particularly, forms a basis at the point of issue the margin of which becomes the seat of evaporation and the consequent precipitation of silica, and is gradually raised by successive deposits of this kind, which also occur on the surrounding surface as the water overflowing the margin spreads itself around. In this way the Great Geyser has heaped up a flat cone of siliceous tufas and sinters to the height of twenty-five or thirty feet, and with a diameter of three hundred. The basin, at its summit, measures at its edge sixty feet in diameter and six or seven feet in depth. The pipe or tube of the basin through which it now receives the water from below is, at its point of junction with the latter, about ten feet in diameter, and has a perpendicular descent of seventy feet.

Iron pyrites has been observed, as a deposit from some thermal waters. Calcareous sulphurets and sulphur, with alumina, are also deposited from thermal sulphureous waters. They make up with baraine what is called mineral mud, which, in proximity to the spring, is used as a kind of bath. Petroleum is occasionally deposited from certain springs, to which Lyell refers the bituminous shales present in geological formations of different ages.

---

A DISH FOR INVALIDS.—Sterne, in a postscript to one of his letters from Montpelier, tells his friend,—"My physicians have almost poisoned me with what they call *bouillons refraichissants;* 'tis a cock flayed alive and boiled with poppy seeds, then pounded in a mortar, and afterward passed through a sieve. There is to be one crawfish in it, and I was gravely told it must be a male one,—a female would do me far more hurt than good."

